# Chloroform and Ether

**Published:** 1894-07

**Authors:** C. G. Darling


### THE DENTAL REGISTER.

Vol. XLVIII.] JULY, 1894. [No. 7.

### Communications.

### Chloroform and Ether.

BY C. G. DARLING, M.D.

Read before the Thirty-eighth Annual Meeting of the Michigan Dental Association.

Nearly half a century has passed away since Wm. G. Morton, an enterprising dentist of Boston, first made use of ether as an anæsthetic, and scarcely less time has elapsed since James Y. Simpson and his friends sat down together to make a practical test of the anæsthetic properties of chloroform.

That these anæsthetics were enthusiastically received and used by the medical and dental professions we can well believe, and that they have so long maintained the place which they now hold in the practice of dentistry when so many local anæsthetics have been brought to notice is a lasting testimony in their favor.

The fact that chloroform was first used in Europe may have influenced the profession of that country to stand so strongly for it and prefer it to ether, but that preference which at one time seemed to be gaining a strong foothold in the United States is fast giving way, and chloroform no longer maintains the place which it once held with ether. That these anæsthetics were at first used indiscriminately, and with little knowledge of the process by which they produced the anæsthetic effect, much less the cause of alarming symptoms, is fully proved by the number of deaths which were soon brought to notice, and the energetic manner in which physicians began to inquire into the cause of death from anæsthesia.

In order that we may judge correctly of the importance of anæsthesia to the profession, I will quote from an edition of Furgeson's Surgery published in 1853.

"A vast and important change has come over the department of operative surgery since the last edition of this work was published. The discovery of anæsthesia as a means of causing insensibility to pain during the performance of surgical operations has been made in the interval, and now instead of wild outcries or stifled screams and groans coming from the patient under the surgeon's instruments, he may be made to lie as quietly as if in a calm sleep, or possibly during the most painful applications of the knife under other circumstances, he may be mentally engaged in the most pleasing associations of thought, or singing or humming by snatches some favorite air."

Still further in this chapter he mentions the dangers and the fact that deaths have already occurred from its use.

The giving of an anæsthetic in those days was entrusted to a man of large experience, one who was able to judge of the condition of patients and the proper method of administering the anæsthetic. The fact that now and then a patient would die while the anæsthetic was being administered led to extensive experiments that some chemical agent might be found to take the place of chloroform and ether, an agent that would be a good anæsthetic but at the same time not dangerous to life; but the work did not meet with good success, not even to the present day. There have been hundreds of deaths reported in those forty-eight years to say nothing of the large number of deaths about which nothing has been said. Lyman has collected three hundred and twenty-three cases of death from chloroform; in thirty-five cases the anæsthetic was administered for tooth extraction, and twenty more died during self-administration. Only twenty-seven deaths from ether had been reported before 1881, none of these for tooth extraction, probably because it was so little employed for that purpose. One hundred and thirty deaths from chloroform were reported from 1880 to 1889. Nine cases were reported for the year 1889 in the journals. One case was in a dental office. The dentist was sued for $5,000, but fortunately for him, the jury disagreed.

Perhaps the latest and most valuable statistics concerning anæsthesia are those reported by Gurlt in the *Archives fur Klinical Chirurg.*, 1893. These cover 50,062 cases of administration with eleven deaths, being one death to every 4,551 cases. All the general anæsthetics, excepting nitrous oxide, are included in this list. The average of chloroform was one death in 2,097 cases, while from ether it was one in 14,646 cases, and this patient died three hours after amputation of both forearms before recovery from the shock of the accident had taken place. The writer concludes his report by saying:

"These quotations leave no doubt that we possess in ether the most harmless and most efficient anæsthetic for all purposes, and that it is our duty wherever human life is concerned to return, when our better judgment tells us, to that anæsthetic with which the era of surgical anæsthesia opened, and to abandon those that have so often endangered life."

Dr. Ormsby, of Dublin, thus graphically describes a death from chloroform which he saw administered: "The man gave but two or three inspirations when he died as suddenly as if shot through the heart. There was no sign of impeded breathing, no pallor of the cheek—in fact, no warning whatever. Never shall I forget the consternation of those concerned! and no wonder, for here was a man in the plenitude of life and apparent vigor, cheerful in spirits, changed, with the appalling suddenness of a lightning flash into a corpse! All means of resuscitation were promptly resorted to but in vain, the man was dead." Yet he tells us that the man had been examined and his heart found perfect but a few moments before the anæsthetic was administered, and the reason assigned for giving chloroform was that he behaved badly under ether which had been administered for some previous operation. This case describes what takes place in a large majority of deaths from chloroform.

The findings of the Hyderabad Commission concerning the use of chloroform have become so well accepted in a general way, that I will take from them freely and make the necessary comments afterward.

The first committee appointed by the Nizam's government, arrived at the conclusion that chloroform arrests the respiration before stopping the heart's action. The *Lancet* would not accept these conclusions, and a second commission was appointed with a representative of the *Lancet* as a member, to test the conclusions of the first and they were found to be correct. The object of this commission, briefly stated, was to test the safety of chloroform in comparison with ether and A. C. E.; the result, if the anæsthetic is pushed to a dangerous degree; the liability to syncope by shock or fatty degeneration of the heart, also the effect of anæsthetics on different animals. While the administration of anæsthetics to animals can hardly be considered a fair test for their effect on man, we will consider their conclusions.

Chloroform freely diluted with air when administered brought about a gradual fall in blood pressure, and the more diluted was the anæsthetic the less rapid was the fall. This fall did not cease when the chloroform had been stopped, because of absorption of residue in the air passages. Respiration may, therefore, cease after the chloroform has been withdrawn. Struggling causes rapid inhalation and, as may be expected, there is rapid fall of blood pressure. The close application of the cone at this time also increases the danger. The controlling action of the vagus upon the heart is its safeguard; it is the temporary exhaustion of the vagus, after stimulation, that proves dangerous. Their experiments give us no new light upon the cause of primary syncope. The value of pulse, color, temperature, pupil and conjunctival reflex, have received little attention at their hands. While fear and shock, such shock as comes only to the human body, largely, it may be, because of anticipation, can not well be studied upon animals, although their experiments contrasted markedly with those occurring in the human subject under similar circumstances. That the danger depends largely upon the dose and that as in other remedies, no two persons require the same dose is probably true; it should then be administered according to the well defined rules which were laid down by the commission, which in substance, are as follows:

1st. The recumbent position on the back and absolute freedom of repose are essential.

2d. That attention to the respiration is necessary to prevent asphyxia when the patient is not in the recumbent position.

3d. If the patient struggles be careful not to interfere, in any way, with respiration when it becomes necessary to hold him down.

4th. Any apparatus which is made to fit the face is an element of danger in the administration of chloroform. And that method of administration only should be used that will admit an abundance of air.

5th. At the commencement of inhalation the cone should be held at such a distance from the mouth or nose as will not cause excitement, struggling, or holding the breath; for if these occur the deep inspirations which follow may cause an overdose. When the patient begins to breathe quickly, the inhaler will be brought closer to the face provided that it does not interfere with respiration. When the patient struggles, cries, or holds the breath, a little fresh air should be admitted, for the deep inspirations which follow, as under such circumstances one or two inhalations of chloroform may be sufficient to produce complete insensibility. The patient is ready for operation when the corneal reflex is abolished, that is, there is no response when the eye is touched with the tip of the finger. He is kept under the anæsthetic by occasional inhalations, and should not be allowed to recover sufficiently to struggle or resist. If we wish to avoid death from shock or fright, the operation should not be commenced until the patient is fully under the influence of the anæsthetic. The administrator should watch the chest and abdomen, and note each respiration. If there is any difficulty of breathing, or the breath is held, the inhaler should be removed until the breathing is again natural. When this does not promptly take place the lower jaw should be pushed forward, the tongue drawn forward, the head lowered. Should this not be sufficient, artificial respiration should be commenced and continued until the breathing is once more natural. Morphia by subcutaneous injection may be given before the anæsthetic in prolonged operations. Alcohol may be given to steady the nerve and increase the confidence.

The Commission ventures the statement that if these rules are strictly followed chloroform may be administered with absolute ease and perfect safety. Without doubt this statement has increased the confidence of the medical profession in the use of chloroform as an anæsthetic, for we find that in the last two years the death rate from this anæsthetic has increased.

The person who is to administer ether should provide himself with a properly constructed cone, one that admits enough air to the respiratory passages to sustain life, not so large an amount as will too much dilute the vapor of ether, as this will greatly retard anæsthesia. When a cone made from cloth and paper is used, the border must be lifted from time to time to admit fresh air. The Allis inhaler is perfectly safe and the amount of air which enters can be regulated by closing the top. The anæsthetizer should provide himself with a forceps for drawing the tongue forward, a gag for separating the jaws, and a mounted sponge for cleansing the pharynx of mucus, for in some cases much mucus will collect. He should have previously examined the heart and lungs to see that they are in a proper condition, and it is always well when there is any suspicion of disease of the kidneys to have the urine examined, because a fatal suppression now and then takes place, especially in cases when the anæsthesia is prolonged. The respiration should not be interfered with in any way, all constricting bands of clothing about the neck and waist should be loosened. The patient should then be laid down upon a couch, table or chair, which will let him assume an easy recumbent posture. No food should be taken for at least four hours before administering ether, because of the liability to cause vomiting. Milk also should be prohibited, as it may add to the danger. It is always well to explain the action of the anæsthetic to the patient, that the first inhalation may cause coughing and a feeling of suffocation, not at all dangerous, and that it will soon wear away. He is instructed to take deep inhalations and to put away all fear.

When the operation is a short one, or the operator works rapidly, primary anæsthesia will suffice, and with a good patient who will obey instructions the operation may be completed in four minutes from the time of beginning the anæsthetic, but the word must be given at the proper moment, or the opportunity is lost until complete anæsthesia is induced. For complete anæsthesia, a large amount of ether is applied to the cone, the patient is instructed to breathe deeply and rapidly, not even a breath of unnecessary air must be allowed to enter, but an atmosphere heavily laden with ether must be rapidly inhaled by the patient. When, however, the operation is quite extensive, full anæsthesia is to be induced. This can be determined by raising the eyelids without resistance or touching the cornea without response. Ether anæsthesia, when complete, will last from two to five minutes, much longer than chloroform, thus giving ample time to perform an extensive extraction without renewing the anæsthesia. It is always well to let the patient become slightly conscious after the first profuse anæsthesia in order that the blood falling back into the mouth may not interfere with respiration. I have seen patients sufficiently aroused to open the mouth when requested and have several teeth drawn afterward, yet declare that they felt no pain. The blood need cause no trouble if the patient's head be turned to one side and a sponge put down between the teeth and cheek to take up the blood as it flows to the lowest part of the mouth.

No one should administer an anæsthetic until he is fully acquainted with the dangers which he may encounter and be prepared to meet them. Death may take place when anæsthesia is complete by the tongue falling back in the throat, thus preventing the entrance of air into the lungs; in this case the tongue should be quickly and forcibly drawn well forward. Again, the muscles of respiration may be convulsed or relaxed. The heart continues to beat but the breathing has ceased. First be sure that the air passages are clear, then begin artificial respiration.

I know no greater danger in ether anæsthesia than that of vomiting large pieces of undigested food, which instead of being ejected may fall back and close the trachea, thus preventing the entrance of air. When the patient attempts to vomit he should be turned to one side and the vomited matter rapidly cleared from the throat; if there should be difficulty of breathing, the lower jaw should be thrust forcibly forward by placing the thumbs behind the angles and pressing forward, this serves to carry the tongue away from the posterior wall of the pharynx.

No person should pin his faith to a single anæsthetic, but be able to judge which will be the better in the case in question. If there is severe cough, bronchitis or any pulmonary obstruction ether should not be used. In advanced arterial disease or for very young children, chloroform should have the preference. Chloroform has generally been stated to be the better anæsthetic for operations about the head and face, but ether is used almost exclusively for such operations in the University Surgical Clinic and with good effect. The operator has much confidence in its safety and is not constantly interrupted to see if his patient is breathing. I have used ether as an anæsthetic a number of times in tooth extraction, and must say that as a rule I much prefer it to chloroform for this purpose: 1st. Because of its safety. 2d. Because anæsthesia lasts much longer when the cone is removed.

The objections raised against ether, viz., the irritation to respiratory organs, the cost, as compared with chloroform, the after-effects, as nausea and vomiting, can scarcely be thought of when we come to consider the safety. Why should we peril human life for the sake of overcoming a few minor inconveniences? If all who are here to-night will give ether a fair trial in dental practice, I have no fear of the result.

